# Molecular and Genetic Research in Tuberculosis Clinical Practice and Epidemiology

**DOI:** 10.5195/cajgh.2014.182

**Published:** 2014-12-12

**Authors:** Bahytkul Zhakipbayeva, Sholpan Beisembayeva, Talgat Muminov

**Affiliations:** Department of Epidemiology, Asfendiyarov Kazakh National Medical University, Almaty, Kazakhstan

**Keywords:** tuberculosis, drug resistance, genotype

## Abstract

**Introduction:**

Tuberculosis (TB) remains a global public health problem. In order for multi-drug resistant tuberculosis (MDR-TB) to be more effectively managed, there is a need for better tools for diagnosis, treatment, and prevention. The decline of TB incidence and mortality in Kazakhstan during last decade was accompanied with consistent growth of MDR-TB. This study aimed to investigate genotype characteristics of Mycobacterium tuberculosis (MT) isolated from TB patients from different regions of the country and its clinical and epidemiological significance.

**Methods:**

Over 500 clinical MT isolates from pulmonary TB patients between 2003–2008 were genotyped using spoligotyping, MIRU-VNTR, IS6110 RFLP, and hybridization on an oligonucleotide biochip “TB–biochip.”

**Results:**

Out of 250 isolates with interpretable results, 31 different spoligopatterns were detected. The Beijing genotype was the most predominant lineage detected (71.6%), characterized by heterogenicity on ETR A, B, C, D, and E markers, and 56.6% of them had an allelic profile 42435. The Beijing genotype and dominating variant strains have a high transmission rate, a high rate of primary MDR (associated with infiltrating lung TB and complications), and a high level resistance to rifampicin and izoniazid due to mutation of rpoB531TTG and katG315ACC. MIRU-VNTR–typing by 15 loci of 33 isolates from 13 family TB foci revealed that strains from supposed sources and contact persons completely coincide in only 5 foci in the genomic structure.

**Conclusion:**

There is a heterogeneous pool of genotypes that circulate in Kazakhstan, with the Beijing lineage being the most predominant. It appears that at the present stage of circulation, MT Beijing genotype has an endemic character. However, clonal spreading of epidemiologically and clinically significant MDR strains of this genotype is also a serious threat to the population. To increase TB control efficiency and prevent further transmission, it is necessary to compile a modern countrywide system of microbiological monitoring for the agent by use of a computer bank of spoligotyping and MIRU-VNTR-typing profiles of circulating strains.

